# The development of a colorectal cancer surgery core outcome set

**DOI:** 10.1186/1745-6215-16-S1-P12

**Published:** 2015-05-29

**Authors:** AGK McNair, RN Whistance, RO Forsythe, R Macefield, J Rees, JE Jones, G Smith, AM Pullyblank, KNL Avery, ST Brookes, MG Thomas, PA Sylvester, A Russell, A Oliver, D Morton, R Kennedy, DG Jayne, R Huxtable, R Hackett, S Dutton, MG Coleman, M Card, J Brown, JM Blazeby

**Affiliations:** 1 Centre for Surgical Research, School of Social and Community Medicine, University of Bristol, Bristol, UK; 2 Division of Surgery Head and Neck, University Hospitals Bristol NHS Foundation Trust, Bristol, UK; 3 Colorectal Cancer Patient Representative, North Bristol NHS Trust, Bristol, UK; 4 Department of General Surgery, North Bristol NHS Trust, Bristol, UK; 5 Severn School of Surgery, Bristol, UK; 6 Colorectal Surgery Unit, University Hospitals Bristol NHS Foundation Trust, Bristol, UK; 7 Colorectal Consumer Liaison Group, National Cancer Research Institute, London, UK; 8 Academic Department of Surgery, University of Birmingham, Birmingham, UK; 9 Department of Surgery, St Mark’s Hospital and Academic Institute, Harrow, UK; 10 Academic Surgical Unit, St James’ University Hospital NHS Trust, Leeds, UK; 11 Centre for Ethics in Medicine, University of Bristol, Bristol, UK; 12 Colorectal Network Site Specific Group, Avon, Somerset & Wiltshire Cancer Services, UK; 13 Centre for Statistics in Medicine, University of Oxford, Oxford, UK; 14 Department of Colorectal Surgery, Plymouth Hospitals NHS Trust, Plymouth, UK; 15 Clinical Trials Research Unit, University of Leeds, Leeds, UK

## Background

Systematic reviews of colorectal cancer surgical trials demonstrate significant heterogeneity of outcome measurement and evidence for selective outcome reporting. This weakens evidence synthesis by hindering meta-analyses, and undermines trial results through outcome reporting bias. This study developed a “core set” of outcomes to be used in trials of colorectal cancer surgery to minimise these limitations.

## Materials and methods

All potential outcomes were identified through systematic literature reviews and interviews with patients. Similar outcomes were grouped into domains and operationalized into a questionnaire survey. Delphi consensus methodology was used to gain agreement between patients, surgeons and nurses as to which outcome domains were “core”. Stakeholders completed questionnaires which asked them to rate the importance of domains on a scale of 1 (not essential) to 9 (absolutely essential). Responses were analysed by retaining outcomes rated between 7-9 by over 50% of respondents and 1-3 by less than 15%. Domains not meeting the pre-defined criteria were discarded after each Delphi round. Domains retained after the second round were brought forward into separate stakeholder meetings to agree on the final core set.

## Results

Data sources identified 1216 outcomes of colorectal cancer surgery that were grouped into 116 domains. A total of 81 UK colorectal cancer centres were surveyed (response rate 79%), including 93 surgeons and 11 clinical nurse specialists, and 97 patients (response rate 36%). 51 outcome domains were retained following the first Delphi survey, and 23 were brought forward to the consensus meetings. Nine outcome domains were considered essential by both stakeholder consensus groups including oncological (long-term survival; cancer recurrence; resection margins), operative (peri-operative survival, surgical site infection, conversion to open surgery (where appropriate), stoma rates and complications) and quality of life (physical and sexual functioning, faecal incontinence and urgency) outcomes.

## Conclusions

This study has developed an evidence-based, internationally ratified core outcome set for colorectal cancer surgery. It is recommended that these outcomes be incorporated into future clinical trials.

